# Jejunal Ectopic Pancreas: A Case Report and Literature Review

**DOI:** 10.7759/cureus.83174

**Published:** 2025-04-29

**Authors:** Logan Prager, Masoumeh Ghayouri, Thomas A Abbruzzese, Ji Fan

**Affiliations:** 1 General Surgery, HCA Florida Hospital Brandon, Brandon, USA; 2 Pathology, Moffitt Cancer Center, Tampa, USA; 3 Gastrointestinal Surgery, Moffitt Cancer Center, Tampa, USA

**Keywords:** ectopic pancreas, heterotopic pancreas, robotic surgery, submucosal lesions, surgical oncology

## Abstract

This case report describes a rare etiology of a symptomatic small bowel mass that had been causing our patient spontaneous abdominal pain, nausea, and loss of appetite. The patient is a 53-year-old Caucasian male undergoing chemotherapy and radiation for a nasopharyngeal Epstein-Barr virus-positive carcinoma. He presented to the urgent care with two days of sharp upper abdominal pain and nausea without association of symptoms to prior chemotherapy treatments. Workup initially suggested a jejunal hematoma, which was later revealed to be a mass. The patient underwent outpatient robot-assisted laparoscopic resection of the mass. Pathology demonstrated benign ectopic pancreatic tissue invading through the serosa into the muscularis propria with the sampled lymph nodes negative for malignancy. Ectopic pancreas is a rare congenital abnormality most commonly found in the gastrointestinal tract. Most cases are asymptomatic, but epigastric pain is the most common symptom, followed by nausea, vomiting, and hematochezia. While a benign finding, the index of suspicion for malignancy should remain high as diagnosis can be difficult. Ectopic pancreatic tissue is susceptible to the same pathology that may affect a native pancreas, including pancreatitis and pancreatic malignancy. Radiological and endoscopy studies are useful adjuncts that may increase clinical suspicion; however, it is difficult to differentiate ectopic pancreas from a neoplasm without tissue biopsy. Although no standardized approach exists, surgical resection remains a mainstay of diagnosis and treatment, especially in anatomic locations not amenable to endoscopic intervention.

## Introduction

Ectopic pancreas is an uncommon phenomenon described as the occurrence of pancreatic tissue in an abnormal anatomic location. This island of pancreatic tissue is completely disconnected from the normal pancreas, with a separate blood supply, innervation, and ductal system. It is most commonly found in the gastrointestinal tract, favoring the stomach and small intestine [1]. However, it can be found anywhere in the abdomen, including the genitourinary system, and more rarely within the thorax [2,3]. While this is an often benign finding, there is a low possibility of malignant conversion [4]. Diagnosis is usually made with pathologic examination after resection, as it may not always appear as a discrete finding on imaging [1]. This case report describes the workup, diagnosis, and treatment of a symptomatic case of ectopic pancreas that initially masqueraded as metastasis of the patient’s active primary cancer.

## Case presentation

The patient is a 53-year-old Caucasian male who was being treated for Epstein-Barr virus-positive nasopharyngeal carcinoma with chemoradiation. His prior history and comorbidities include diabetes mellitus, hyperlipidemia, hypertension, and a 30-pack-year smoking history with successful cessation seven years prior. The patient received his cancer diagnosis after noticing an enlarged right post-auricular lymph node and subsequently receiving a computed tomography scan of the neck identifying enlarged lymph nodes in the right retropharyngeal space with poorly defined borders associated with a 3.7 cm right nasopharyngeal mass without invasion of the skull base. Combined positron emission tomography and computed tomography scan confirmed metabolically active lymph nodes in the bilateral neck. There were no abnormalities appreciated within the abdomen. The nasopharyngeal mass was biopsied with resultant pathology demonstrating nasopharyngeal carcinoma, undifferentiated, non-keratinizing, Epstein-Barr virus-encoded small RNAs (EBER) positive, and negative for p16 expression.

He presented to our facility’s urgent care with complaint of constipation and sharp left upper quadrant pain which radiated to the left upper quadrant. He attempted relief of this new onset pain with oxycodone previously prescribed for his nasopharyngeal cancer-related pain limited to the head and neck. However, this had little therapeutic effect and he discontinued use one day prior to presentation due to concern of worsening constipation. He reported poor appetite due to persistent nausea after each ingestion of food or drink. Additionally, he had chills but denied fevers. He continued to pass flatus throughout the course of these symptoms without blood or mucus in his stool. The increased severity of this episode of symptoms prompted him to seek care.

The patient’s physical exam was largely nonspecific, with tenderness appreciated from the left upper quadrant to the right upper quadrant in a band-like fashion. Otherwise, the abdomen showed no signs of peritonitis and he was comfortable, well-appearing, and fully oriented. His vital signs and temperature were within the normal range and remained stable. The remainder of the physical exam revealed no abnormal findings.

The patient underwent extensive evaluation with laboratory testing and imaging at the initial presentation. Lab work was unrevealing and grossly normal, with no significant abnormalities appreciated on complete blood count, chemistry panels, or coagulation studies. Preliminary imaging with a computed tomography scan of the abdomen and pelvis revealed a focus of thickened and edematous jejunal wall with intramural hyperdensity and associated mesenteric stranding within the left upper quadrant. These findings were suspicious for an intramural hematoma or hemorrhage, with metastatic, infectious, and inflammatory processes lower on the list of differential diagnosis. The patient did not have any recent trauma, signs, or symptoms of bleeding. Of note, he had received platinum-based chemotherapy eight weeks prior to presentation which uncommonly causes ulceration and inflammation of the bowel and colon. Further investigation was needed to rule out metastasis from his nasopharyngeal cancer as well as enteritis from platinum-based chemotherapy. Repeat CT scan with IV contrast three days after initial presentation redemonstrated the findings from the initial study, in addition to nodular thickening of the jejunal serosal surface and adjacent mesentery with prominent mesenteric, periportal, and portocaval lymph nodes. At this time, his abdominal pain, nausea, and vomiting had resolved with bowel rest, and he was maintaining tolerance of a regular diet with normal non-bloody bowel movements. He was discharged with follow-up in clinic and repeat imaging two weeks from discharge for evaluation of the possible hematoma versus mass. The patient had no recurrence of symptoms in the outpatient setting after resolution of them prior to discharge. The third and fourth CT scans with IV contrast performed in the outpatient setting showed persistence of the jejunal serosal lesion which gave more credence to the lesion being a mass rather than a hematoma with a concern for metastasis from his nasopharyngeal carcinoma or second primary neoplasm. Enteroscopy was considered to obtain tissue diagnosis but ultimately not performed, as the jejunal lesion was deemed too far to reach for biopsy. At this time, our team discussed our recommendation for surgical resection with the patient, who agreed to proceed with a robot-assisted small bowel resection for both diagnostic and therapeutic intent.

The definitive intervention selected was a robot-assisted small bowel resection. Gross evaluation identified the lesion to be a solid mass involving the wall of the jejunum and was about 60 cm distal to the ligament of Treitz. The operation proceeded successfully with removal of the small bowel mass en bloc with an approximately 15 cm affected loop of jejunum and creation of a primary jejunojejunal anastomosis. Indocyanine green dye was used to confirm that the anastomosis was well-perfused. No obvious carcinomatosis was noted intra-operatively upon surveillance of the peritoneum. 

The patient had an uneventful post-operative course and was discharged on post-operative day two after tolerance of diet and passing flatus. He had a normal post-operative follow-up and no recurrence of presenting symptoms.

**Figure 1:**
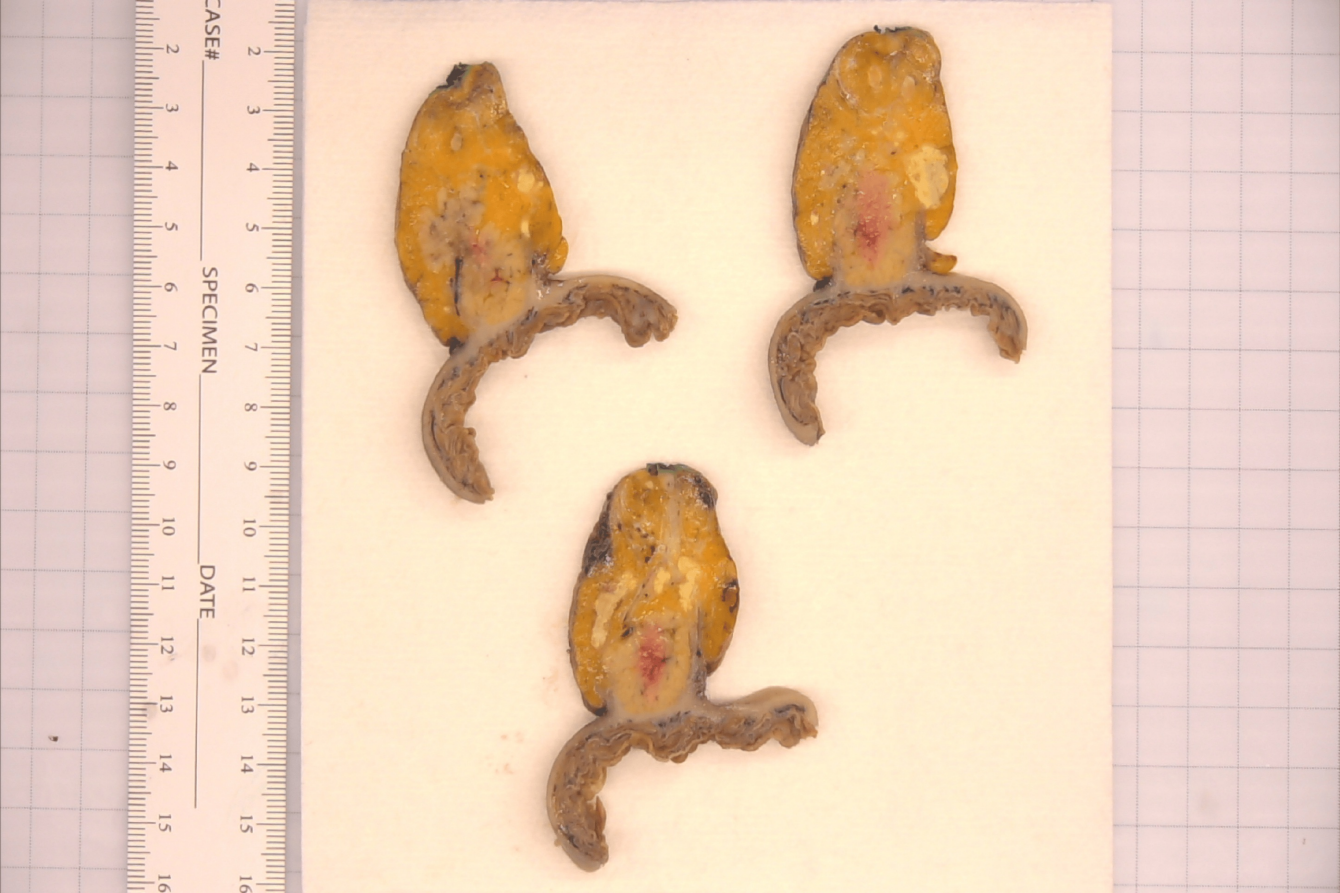
Gross Pathology Ectopic pancreas invading the serosa of the jejunum present at the inferior aspect of each specimen. Central hemorrhage within the heterotopic pancreatic tissue and caseating granulomas within the surrounding mesenteric fat.

**Figure 2:**
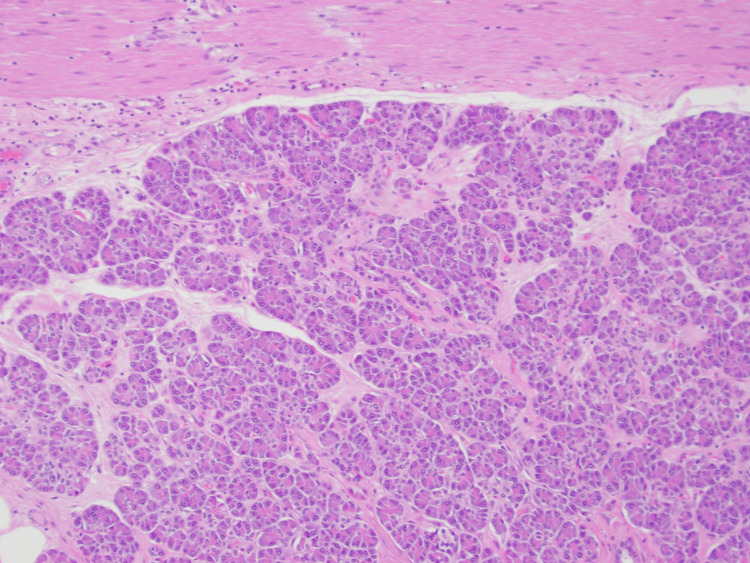
10x Microscopic View of Specimen Normal pancreatic histological architecture of acinar cells and intralobular ducts with jejunal serosal cells present at the top of the image.

The pathology revealed benign heterotopic pancreatic tissue involving the serosa of the jejunum and infiltrating the adjacent mesentery of approximately 3.5 cm in the largest dimension (Figure 1). Additionally, there were multiple granulomas with central necrosis present in the surrounding fat, which may have been related to exocrine function from the ectopic pancreatic tissue. There was no evidence of malignancy on microscopic evaluation, with nine negative sampled lymph nodes and microscopic margins clear of dysplasia or malignancy (Figure 2).

## Discussion

Ectopic pancreas is defined as the presence of pancreatic tissue outside its typical location and without vascular or anatomic connections to the pancreas. It was first described in 1727, found within an ileal diverticulum. It is thought to be a congenital anomaly of migration that is not currently completely understood. The prevailing explanation is called dislocation or misplacement theory, whereby pancreatic tissue separates from the original pancreas and deposits in another area during foregut rotation [1]. Other explanations include metaplastic theory, suggesting that endodermal pancreatic metaplasia migrates to the submucosa during embryogenesis, or ectopic pancreatic tissue arising from teratomas present in remote sites [4]. These areas of deposition are predominantly found throughout the gastrointestinal tract, but there have been reported cases of ectopic pancreatic tissue being found in more unusual places such as the biliary system, liver, fallopian tube, lung, mediastinum, and brain [2-4]. The most common areas to find ectopic pancreatic tissue are the proximal gastrointestinal tract including the stomach (25-62%), duodenum (25-35%), and jejunum (16%) [4].

Ectopic pancreas is a relatively rare condition affecting an estimated 0.5% to 13% of people in the general population [1]. It is often asymptomatic, occult, or incidentally found on imaging, but may present with symptoms consisting of nonspecific abdominal pain, nausea, and vomiting as was seen in this case [4]. However, more severe pathology may occur, including enteroenteric or enterocolonic intussuception [5], gastric outlet or small bowel obstruction, hollow viscus perforation [6], pancreatitis [7], gastrointestinal bleeding [8], or even malignant transformation [4].

Regarding the possibility of malignancy, transformation of benign ectopic pancreatic tissue is thought to be rare with an incidence ranging from 0.7 to 1.8% of cases [4]. To be considered malignant transformation, the tumor must be within or near the ectopic pancreatic tissue, have an observable transition from pancreatic tissue to carcinoma, invasion from an independent malignancy must be excluded, and the benign pancreatic tissue must display fully developed acini and ductal structures. In a systematic review of published cases of malignant transformation of ectopic pancreas, several associations have been described [4]. The mean age for patients with this condition was 55.6 years old with a slight male predominance of 51.8% of cases, consistent with our patient's demographics. The majority of patients with malignant transformation were symptomatic (83%). The majority of cases were located within the stomach (35.2%), duodenum (22.2%), and jejunum (14.8%), consistent with the reported distribution of incidence of ectopic pancreas [4].

Diagnosis of ectopic pancreas is notoriously difficult from imaging studies alone. This may be due to variance in presentation on cross-sectional imaging and inability to reach the lesion with the ideal imaging modality of endoscopic ultrasound. Intravenous contrast-enhanced computed tomography scan is useful in locating ectopic pancreatic tissue as it usually presents as an enhancing submucosal mass similar to a leiomyoma or carcinoid tumor, however hemorrhagic or inflammatory components may limit the utility of this modality [9]. On endoscopic evaluation, a submucosal mass may be appreciated growing into the lumen of the affected organ. Endoscopic ultrasound can better delineate the morphology of these ectopic pancreatic masses and also determine where they originate, most commonly found in the submucosa or muscular layers [1]. As an adjunct, a fine needle aspiration can be performed to obtain a tissue diagnosis, and multiple passes are recommended due to the heterogeneity of the tissue sampled in order to get an accurate diagnosis. 

Treatment of these lesions spans from observation to surgical resection. The majority of asymptomatic ectopic pancreatic masses can be observed once malignancy is ruled out. Minimally invasive options exist utilizing endoscopic resection. These include submucosal dissection, submucosal tunnel endoscopic resection, high-frequency resection, mucosal resection, full-thickness resection, or ligation in some cases [1]. However, if the lesion cannot be reached endoscopically or it involves the entire wall of the affected organ, or if there is high suspicion for malignancy, such as in our patient’s case, then surgical resection is indicated [1]. En bloc resection of the ectopic pancreatic tissue either by surgical or endoscopic excision is curative and provides enduring relief of symptoms.

## Conclusions

Ectopic pancreas is a rare congenital anomaly with varying anatomic locations and clinical presentations. It is often benign and asymptomatic, but may present anywhere on a spectrum of severity ranging from mild nonspecific abdominal pain to more worrisome complications like bowel obstruction or bleeding. Despite this benign growth mimicking a local malignancy, there is a possibility of true malignant transformation of the heterotopic pancreatic tissue. Accurate diagnosis is crucial and reliably obtained with tissue biopsy. Surgical resection is the definitive management for lesions too large for or inaccessible via endoscopy and provides the opportunity for both symptomatic relief and confirmatory pathology. 
